# Attainment of therapeutic vancomycin level within the first 24 h

**DOI:** 10.1186/s13054-019-2515-5

**Published:** 2019-06-21

**Authors:** Patrick M. Honore, David De Bels, Rachid Attou, Sebastien Redant, Andrea Gallerani, Kianoush Kashani

**Affiliations:** 1 ICU Department, Centre Hospitalier Universitaire Brugmann, Brugmann University Hospital, 4 Place Arthur Van Gehuchten, 1020 Brussels, Belgium; 2 Division of Nephrology and Hypertension, Division of Pulmonary and Critical Care Medicine, Mayo Clinic, Rochester, USA

Attaining adequate vancomycin levels among critically ill patients is an important issue. The early achievement of this target potentially avoids therapy failure, the emergence of multiresistant bacterial strains, and even possibly increased mortality [1, 2]. The primary goal of the study by Batista and colleagues [1], who used a creatinine clearance (ClCr)-based nomogram, was reaching the vancomycin target level with continuous infusion (CI) strategy. The authors reported no additional risk of nephrotoxicity and found CI to be superior to a loading dose of 35 mg/kg [1, 3]. Augmented renal clearance (ARC; creatinine clearance > 130 ml/min) was present in 40% of critically ill patients without acute kidney injury. The target vancomycin level of 20–30 mg/L at 24 h was achieved in 84% of patients [1]. Cristillini et al., in a cohort with a 21% incidence of ARC, used a loading dose of 35 mg/kg in 4 h and achieved the same target vancomycin (at 24 h) trough levels in 54% of the patients [4]. Vancomycin dosing during continuous renal replacement therapy (CRRT) is challenging and yet very important. Beumier et al. described that a 35-mg/kg loading dose during CRRT allows therapeutic vancomycin target level attainment in 63% of patients within 24 h [5]. Unfortunately, dosing nomogram proposed by Batista et al. has not been assessed in patients treated with RRT [1]. At this point, considering the lack of data, the loading vancomycin dose of 35 mg/kg remains the gold standard in CRRT patients. As there is a critical need to ascertain the most effective strategy to attain vancomycin therapeutic trough levels in patients treated with CRRT, future studies need to compare Batista et al. dosing protocol with the current standard of care.

## Data Availability

Not applicable.

## Abbreviations

ARC: Augmented renal clearance
CI: Continuous infusion
ClCr: Creatinine clearance
CRRT: Continuous renal replacement therapy
ICU: Intensive care unit
RRT: Renal replacement therapy
